# A systematic review and meta-analysis of treatment-related toxicities of curative and palliative radiation therapy in non-small cell lung cancer

**DOI:** 10.1038/s41598-021-85131-7

**Published:** 2021-03-15

**Authors:** M. Or, B. Liu, J. Lam, S. Vinod, W. Xuan, R. Yeghiaian-Alvandi, E. Hau

**Affiliations:** 1 Department of Radiation Oncology, The Crown Princess Mary Cancer Centre, Westmead Hospital, Westmead Sydney, NSW 2145 Australia; 2 Northern Sydney Cancer Centre, Royal North Shore Hospital, Sydney, NSW Australia; 3 Cancer Therapy Centre, Liverpool Hospital, Liverpool, NSW Australia; 4 South Western Sydney Clinical School, University of New South Wales, Sydney, NSW Australia; 5 Ingham Institute for Applied Medical Research, Liverpool, NSW Australia

**Keywords:** Non-small-cell lung cancer, Radiotherapy

## Abstract

Treatment-related toxicity is an important component in non-small cell lung cancer (NSCLC) management decision-making. Our aim was to evaluate and compare the toxicity rates of curative and palliative radiotherapy with and without chemotherapy. This meta-analysis provides better quantitative estimates of the toxicities compared to individual trials. A systematic review of randomised trials with > 50 unresectable NSCLC patients, treated with curative or palliative conventional radiotherapy (RT) with or without chemotherapy. Data was extracted for oesophagitis, pneumonitis, cardiac events, pulmonary fibrosis, myelopathy and neutropenia by any grade, grade ≥ 3 and treatment-related deaths. Mantel–Haenszel fixed-effect method was used to obtain pooled risk ratio. Forty-nine trials with 8609 evaluable patients were included. There was significantly less grade ≥ 3 acute oesophagitis (6.4 vs 22.2%, p < 0.0001) and any grade oesophagitis (70.4 vs 79.0%, p = 0.04) for sequential CRT compared to concurrent CRT, with no difference in pneumonitis (grade ≥ 3 or any grade), neutropenia (grade ≥ 3), cardiac events (grade ≥ 3) or treatment-related deaths. Although the rate of toxicity increased with intensification of treatment with RT, the only significant difference between treatment regimens was the rate of oesophagitis between the use of concurrent and sequential CRT. This can aid clinicians in radiotherapy decision making for NSCLC.

## Introduction

Lung cancer remains the leading cause of cancer mortality worldwide^1^, the majority of lung cancer is non-small cell lung cancer (NSCLC)^2^. For patients with unresectable NSCLC, radiation therapy (RT) treatment options include concurrent chemoradiation (CRT), sequential CRT, curative RT and palliative RT.

Although the treatment regimen that provides the highest cure rate for each disease stage is well established, population studies have shown that treatment in NSCLC is consistent with guidelines in only 44–52% of cases^3–5^, and radiotherapy remains underutilised across the world^6^. While many factors influence the under-utilisation of radiotherapy, an important aspect is clinician concern regarding treatment-related toxicity, where treatments associated with better survival outcomes have increased toxicity. Comorbidity potentially influencing treatment is prevalent in 72%-81% of lung cancer patients^7–9^. This has been associated with reduced likelihood of patients receiving radiotherapy^7^.

Numerous studies now reported on survival prediction models for NSCLC, two from the MAASTRO group^10,11^. These both show that even with curative radiotherapy (± chemotherapy), there are different prognostic groups of patients, some who do poorly despite radical RT and some who do well. If clinicians are to use these models then the patient also needs to be informed of toxicity predictions for shared decision making. Some ‘poor risk’ patients may choose to accept higher toxicity rates with curative RT despite small survival gains, and others may not. However, the available literature can be difficult to interpret when quantifying the rate of toxicity between different treatment regimes. Due to the variable toxicity types and rates that is reported in individual trials, better estimates of toxicities would be helpful in guiding clinical management.

The aim of this systematic review and meta-analysis is to evaluate and statistically combine toxicity rates of curative and palliative RT (excluding stereotactic body radiation therapy) with or without chemotherapy for patients with unresectable NSCLC. This information increases the precision of the quantitative estimates of the toxicity rates compared to individual trials.

## Methods

A systematic search of electronic databases (MEDLINE, PubMed, EMBASE, and the Cochrane Central Register) was performed using the following terms: non-small cell lung cancer, radiation therapy, radiotherapy, randomised controlled trial, controlled clinical trial, controlled trials, systematic review, and meta-analysis. We included recent studies published between January 2000 and June 2019. Searches were limited to human studies published in English. When multiple studies of the same clinical trial were encountered, the updated results were included. The PRISMA guidelines (Preferred Reporting Items for Systematic Reviews and Meta-Analyses) were used to assist in writing this review^12^.

References identified by the search strategy were screened independently by two investigators (M.O. and B.L.) to review the trials for eligibility for inclusion and the list of trials eligible for inclusion was agreed.

### Inclusion and exclusion criteria

Studies that met the following criteria were included: published randomised trial with greater than 50 patients with unresectable NSCLC undergoing curative and/or palliative RT. Curative RT was defined as a minimum dose of 50 Gy, or its radiobiological equivalent, with or without chemotherapy^13^. Palliative RT was defined as a dose of less than 50 Gy. Unresectable disease could be medically or surgically inoperable.

We excluded trials with small cell lung cancer or recurrent lung cancer. Patients treated with prior high dose RT in region of lung cancer, stereotactic body radiation therapy (SBRT), protons, carbon-ions, post-operative RT or palliative CRT were also excluded.

### Data extraction

The details of included trials were recorded independently by two authors (M.O and B.L) via a data collection template (Appendix 1). Any discrepancy was resolved by consensus with third party (J.L.). Patient and trial characteristics, including disease stage, median age, study type, follow-up and toxicity criteria used were extracted along with summary information on treatment characteristics (treatment regime, chemotherapy type and timing, dose and fractionation). Treatment-related toxicity for each RT regimen was extracted, including the incidence and grade of oesophagitis, pneumonitis, cardiac events, pulmonary fibrosis, radiation myelopathy, neutropenia and/or treatment-related death (TRD).

### Statistical analysis

The pooled risk of toxicities by any grade, grade ≥ 3, and treatment related deaths were expressed as the total number of cases for each toxicity outcome divided by the total number of patients treated with the same type of treatment. Treatment regimens were categorised into palliative RT alone, curative RT alone, sequential CRT and concurrent CRT. We performed indirect comparisons to estimate the risk ratio for the comparison between palliative versus curative RT and sequential versus concurrent CRT. The Mantel–Haenszel fixed-effect method was used to obtain the pooled risk ratio and corresponding confidence interval. We used the fixed-effect method for all comparisons for consistency. Statistical heterogeneity was assessed by calculating I^2^. Cochrane Review Manager version 5.3 (Cochrane Collaboration, Copenhagen, Denmark) was used for the analyses.

### Quality assessment

The risk of bias for each trial was assessed using the criteria outlined in the Cochrane Handbook for Systematic Reviews of Interventions^14^. These include random sequence generation, allocation concealment, incomplete outcome data, selective reporting and other biases (such as method of assessing symptoms).

## Results

### Eligible studies

**Figure 1:**
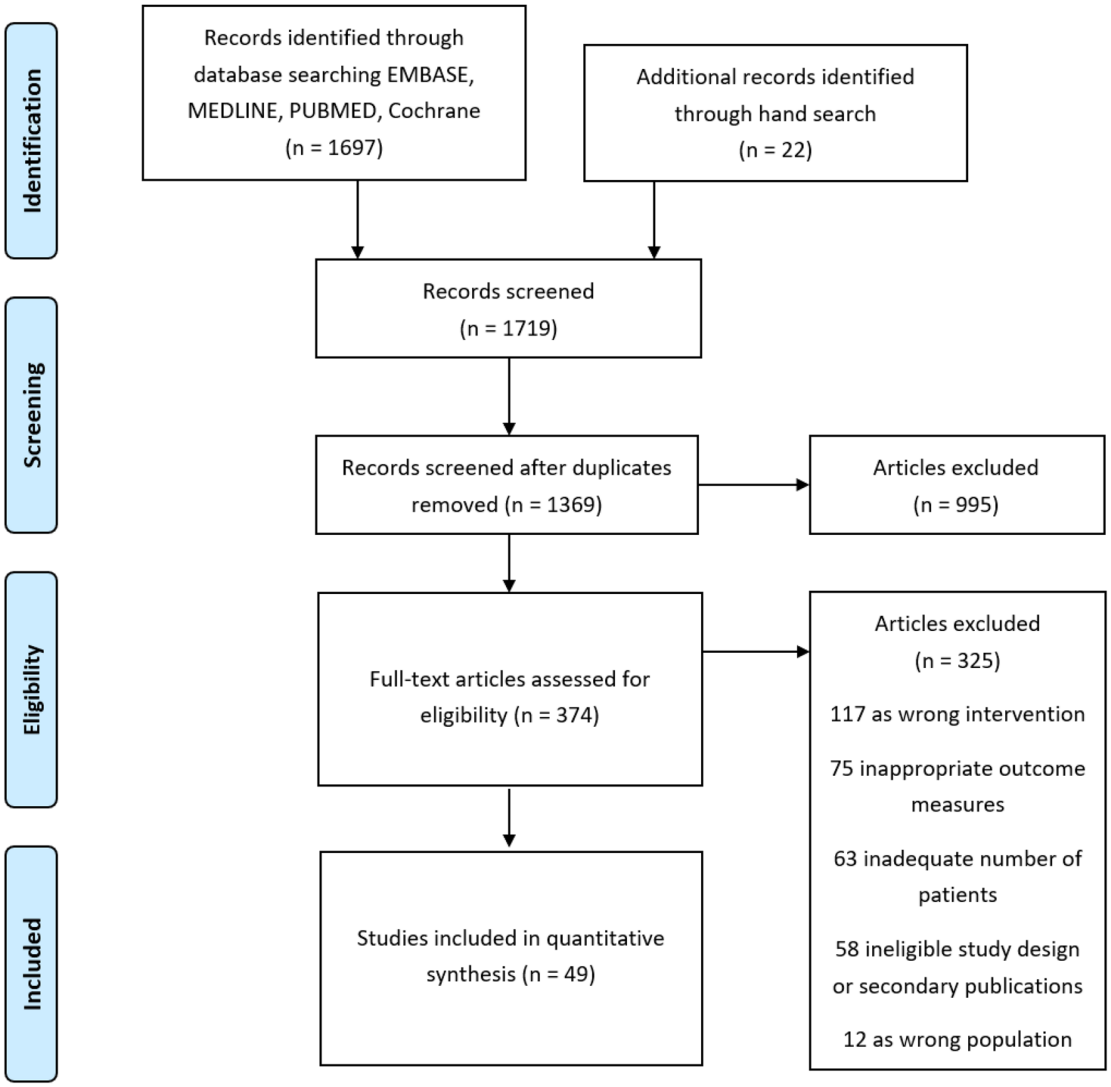
PRISMA Flow diagram^12^ with details of the number of studies identified, screened, assessed and included in the final review.

We identified 49 eligible trials^15–64^ with a total of 10,388 patients, of which 8609 were evaluable for toxicity (Fig. 1). The overall trial characteristics are shown in Table 1. There was variability in the reporting of symptoms, with various versions of 5 different toxicity grading criteria used in 39 of the included trials. 8 trials included stage IV patients accounting for 1835 patients. 5 of these were palliative trials and the remaining 3 trials only had a small proportion (23 patients) of stage IV disease. Treatment characteristics of included trials are summarised in Table 2 demonstrating the heterogeneity with respect to the study design, toxicity scoring criteria, treatment arms, RT dose fractionation and chemotherapy regimen. There was a wide range of RT dose fractionation used, from 10 Gy in 1 fraction for palliative RT, up to 74 Gy in 37 fractions in concurrent CRT. Most chemotherapy regimens were platinum-based. 2 studies assessed elderly patients^17,18,60^.

**Table 1:** Patient and study characteristics of included trials.

Author (year)	N (evaluable)	Stage	Median age	Median follow-up (months)	Toxicity criteria	Risk of bias
Antonadou (2002)	191 (96)	IIb–IV	Mean 65*	–	RTOG	High
Antonia (2017)	713 (234)	III	64	14.5	CTCAE v4	High
Atagi (2012)†	200 (197)	III	77	19 (†108)	NCI-CTC v2	Some concerns
Ball (2019)	101 (35)	I	77	25.2	CTCAE v4	High
Belani (2005a)	141 (113)	III	63–66	20.3	CTCAE v2	High
Belani (2005b)	276 (256)	III	24% ≥ 70 years*	39.6	NCI-CTC and RTOG	High
Belderbos (2007)	158 (142)	I–III	62–64	39	RTOG	High
Bezjak (2002)	230 (230)	III–IV	70	–	NCI CTG expanded CTC	High
Bradley (2015)	544 (258)	III	64	22.9	CTCAE v3	High
Cakir (2004)	185 (176)	III	60–61	–	WHO	High
Crvenkova (2018)	85 (85)	III	Range 18–70*	–	RTOG/EORTC	High
Curran (2011)	610 (575)	II–III	61	132	–	High
Edelman (2017)	60 (22)	IIIa	61	–	CTCAE v4	Some concerns
Erridge (2005)	149 (126)	–	Mean 66–68*	Follow-up until death	–	High
Fairlamb (2005)	288 (115)	I–IV	64	39.5	–	High
Falk (2002)	230 (230)	–	71	–	–	Some concerns
Feng (2016)	72 (36)	III	63	–	CTCAE v3	Some concerns
Fournel (2005)	205 (193)	III	56–57	57.6	WHO	High
Fournel (2016)	127 (127)	III	57–59	76.8	CTCAE v2	High
Gouda (2006)	60 (60)	III	59–62	–	RTOG	High
Hanna (2008)	203 (147)	III	63	41.6	CTCAE v3	High
Hansen (2017)	117 (117)	IIb–III	65–67	32.6	CTCAE v3	High
Huber (2006)	219 (212)	III	Mean 62*	13.6	WHO	High
Jalal (2012)	243 (243)	III	26% ≥ 70 years*	–	–	High
Johnstone 2002)	73 (32)	IIIa	–	–	–	High
Kelly (2008)	571 (543)	III	61	27	NCICTC v2	High
Kramer (2005)	297 (297)	III–IV	69	–	NCI CTG expanded CTC	High
Lee (2017)	59 (52)	III	60–62	23.6 (Surviving patients)	CTCAE v3	High
Liang (2017)	191 (191)	III	57–59	73	NCICTC v3	High
Lu (2010)	379 (191)	III	63	44.4	CTCAE v2	High
Movsas (2010)	64 (64)	III	59	41.5	CTCAE v2	High
Nawrocki (2010)	99 (48)	III	66	41	CTCAE v2	High
Nestle (2000)	152 (152)	III–IV	66	12	RTOG	High
Nyman (2016)	102 (53)	I	Mean 74*	37	CTC v3	Some concerns
Pan (2016)	117 (117)	IIb–III	66	–	CTCAE v3	Some concerns
Reinfuss (2005)	173 (173)	III	> 58	Minimum 12	RTOG	High
Sasaki 2018)	108 (108)	III	60–62	31.9	CTCAE v3	Low
Scagliotti (2006)	89 (87)	III	59	-	CTCAE	High
Sculier (2018)	125 (120)	III	57–60	62	WHO	High
Senan (2016)	598 (555)	III	59–60	22 -23	CTCAE v3	High
Senkus-Konefka (2005)	100 (98)	III–IV	66–67	–	–	High
Shibamoto (2001)	301 (101)	III	N/A	–	RTOG	High
Su (2019)	101 (101)	IV	< 60	–	CTCAE v3	Some concerns
Sundstrom (2004)	421 (407)	III–IV	68–69	Follow-up until death	–	Some concerns
Takigawa (2011)	200 (199)	III	< 70	–	CTCAE v2	High
van Diessen (2019)	107 (77)	II–III	64	38	CTCAE v3	Some CONCERNS
Vokes (2002)	187 (175)	III	61	43	–	High
Yamamoto (2010)	456 (440)	II–IV	62–63	Follow-up period 36	–	High
Zatloukal (2004)	102 (99)	III	62	Minimum 18	WHO	High

*Median not reported.

^†^Updated in 2018, includes censored cases.

– Not available.

Belani (2005a)—ECOG 2597.

Belani (2005b)—Combined chemoradiotherapy regimens of paclitaxel and carboplatin for locally advanced non-small-cell lung cancer: A randomized phase II locally advanced multi-modality protocol.

**Table 2:** Treatment characteristics of included trials.

Author (year)	Treatment arm(s)	SCRT chemotherapy regimen	CCRT chemotherapy regimen	Radiation dose fractionation
Antonadou (2002)	CCRT, SCRT, cRT	Platinum based	–	55–60 Gy/27–30# ± 5-10 Gy Boost
Antonia (2017)	CCRT	–	Platinum based	54–60 Gy/27–30#
Atagi (2012)^†^	CCRT, cRT	–	Carboplatin	60 Gy/30#
Ball (2019)	cRT	–	–	66 Gy/33# or 50 Gy/20#
Belani (2005a)	SCRT	Carboplatin/paclitaxel	–	64 Gy/32# or 57.6 Gy/36# TDS
Belani (2005b)	CCRT, SCRT	Carboplatin/paclitaxel	Carboplatin/paclitaxel	63 Gy/34#
Belderbos (2007)	CCRT, SCRT	Cisplatin/gemcitabine	Cisplatin	66 Gy/24#
Bezjak (2002)	pRT	–	–	20 Gy/5# or 10 Gy/1#
Bradley (2015)	CCRT	Carboplatin/paclitaxel	Carboplatin/paclitaxel	74 Gy/37# or 60 Gy/30#
Cakir (2004)	CCRT, cRT	–	Cisplatin	64 Gy/32#
Crvenkova (2018)	CCRT, SCRT	Carboplatin/paclitaxel, carboplatin/etoposide	Cisplatin/etoposide	60 Gy/30#
Curran (2011)	CCRT, SCRT	Cisplatin/vinblastine	Cisplatin/etoposide or cisplatin/Vinblastine	69.6 Gy/58# BD or 60 Gy/30#
Edelman (2017)	CCRT	Carboplatin	Carboplatin/paclitaxel	60 Gy/30#
Erridge (2005)	pRT	–	–	30 Gy/10# or 10 Gy/1#
Fairlamb (2005)	CCRT, SCRT	Cisplatin based	–	50–55 Gy/20#
Falk (2002)	pRT	–	–	17 Gy/2# weekly or 10 Gy/1#
Feng (2016)	CCRT	–	Cisplatin	60 Gy/30#
Fournel (2005)	CCRT, SCRT	Cisplatin/vinorelbine	Cisplatin/etoposide	66 Gy/33#
Fournel (2016)	CCRT	Cisplatin/paclitaxel	Cisplatin/vinorelbine	66 Gy/33#
Gouda (2006)	CCRT, cRT	Carboplatin/paclitaxel	Carboplatin/paclitaxel	60 Gy/30#
Hanna (2008)	CCRT	Docetaxel	Cisplatin/etoposide	59.4 Gy/33#
Hansen (2017)	CCRT	Carboplatin/vinorelbine	Vinorelbine	66 Gy/33# or 60/30#
Huber (2006)	CCRT, SCRT	Carboplatin/paclitaxel	Paclitaxel	60–66 Gy/30–33#
Jalal (2012)	CCRT	Docetaxel	Cisplatin/etoposide	59.4 Gy/33#
Johnstone 2002)	SCRT	Cisplatin/mitomycin-C±vinblastine	–	64 Gy/32#
Kelly (2008)	CCRT	Docetaxel	Cisplatin/etoposide	61 Gy/33#
Kramer (2005)	pRT	–	–	30 Gy/10# or 16 Gy/2# weekly
Lee (2017)	CCRT	Cisplatin/Irinotecan	Cisplatin/irinotecan	60 Gy/30#
Liang (2017)	CCRT	–	Cisplatin/etoposide or carboplatin/paclitaxel	60–66 Gy/30–33#
Lu (2010)	CCRT	Carboplatin, cisplatin/vinorelbine	Carboplatin/paclitaxel or cisplatin/vinorelbine	60 Gy/30#
Movsas (2010)	CCRT	Gemcitabine, gemcitabine/docetaxel	Cisplatin/etoposide	62 Gy/31#
Nawrocki (2010)	pRT	–	–	30 Gy/10#
Nestle (2000)	cRT, pRT	–	–	60 Gy/30# or 32 Gy/16# BD
Nyman (2016)	cRT	–	–	70 Gy/35#
Pan (2016)	CCRT	Carboplatin/vinorelbine	Vinorelbine	66 Gy/33# or 60 Gy/30#
Reinfuss (2005)	CCRT, SCRT	Cisplatin/navelbine	Cisplatin/navelbine	70.2 Gy/39#
Sasaki 2018)	CCRT	–	Cisplatin/S1 or cisplatin /vinorelbine	60 Gy/30#
Scagliotti (2006)	CCRT, SCRT	Cisplatin/docetaxel	Docetaxel	60 Gy/30#
Sculier (2018)	CCRT	Cisplatin/docetaxel	Cisplatin/docetaxel	66 Gy/33#
Senan (2016)	CCRT	Platinum based doublet, premetrexed	Cisplatin/etoposide or cisplatin/pemetrexed	60–66 Gy/30–33#
Senkus-Konefka (2005)	pRT	–	–	20 Gy/5# or 16 Gy/2# weekly
Shibamoto (2001)	CCRT	–	Carboplatin/etoposide	69.6 Gy/58# BD
Su (2019)	CCRT	–	Cisplatin/premetrexed or cisplatin/docetaxel	40 Gy/20# + 20–30 Gy/1.5 Gy BD
Sundstrom (2004)	cRT, pRT	–	–	50 Gy/25# or 42 Gy/15# or 17 Gy/2#
Takigawa (2011)	CCRT	–	Cisplatin/docetaxel or cisplatin/mitomycin/vindesine	60 Gy/30#
van Diessen (2019)	CCRT	–	Cisplatin based	≥ 72 Gy/24#
Vokes (2002)	CCRT	Cisplatin/gemcitabine, paclitaxel, vinorelbine	Cisplatin based	66 Gy/33#
Yamamoto (2010)	CCRT	Platinum based	Platinum based	60 Gy/30#
Zatloukal (2004)	CCRT, SCRT	Cisplatin/vinorelbine	Cisplatin/vinorelbine	60 Gy/30#

^†^Updated in 2018, includes censored cases.

Not available.

Belani (2005a)—ECOG 2597.

Belani (2005b)—Combined Chemoradiotherapy Regimens of Paclitaxel and Carboplatin for Locally Advanced Non-Small-Cell Lung Cancer: A Randomized Phase II lLocally Advanced Multi-Modality Protocol.

pRT—Palliative Radiotherapy.

cRT—Curative Radiotherapy without chemotherapy.

SCRT—Sequential chemoradiotherapy (induction or consolidation chemotherapy).

CCRT—Concurrent chemoradiotherapy (with or without sequential chemotherapy).

### Treatment-related death

**Figure 2:**
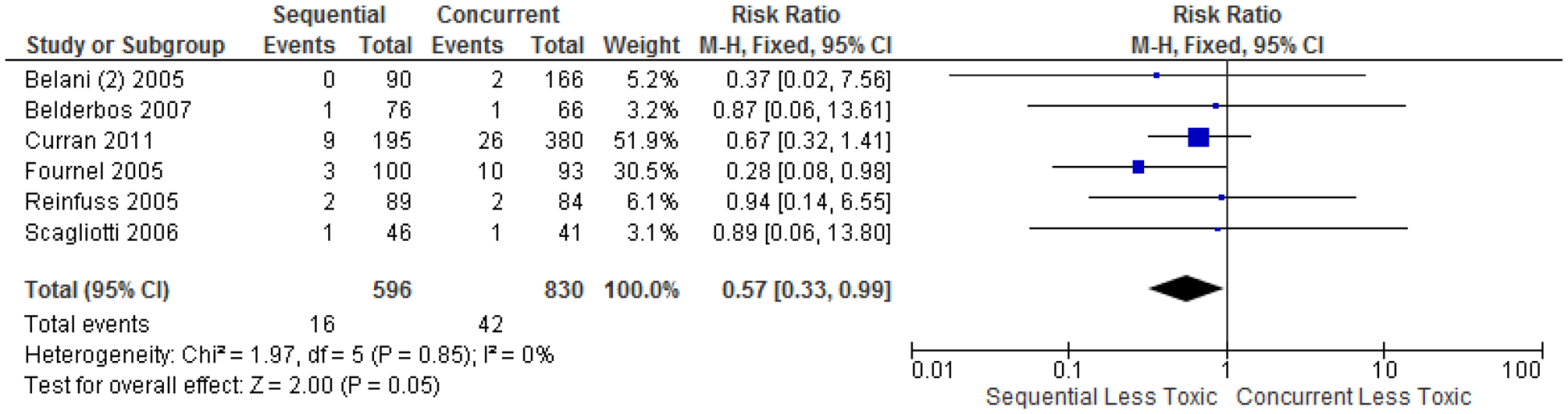
Forest plot showing toxicity risk ratio (RR) for treatment-related death; comparison between sequential versus concurrent chemoradiation, generated with Cochrane Review Manager version 5.3

The overall rate of TRD was low on indirect comparisons, highest in concurrent CRT (3.1%), followed by sequential CRT (2.3%), curative radiation alone (2.4%) and palliative radiation (0%). In the 6 trials^20,22,27,33,51,53^ that directly compared concurrent with sequential CRT, TRD from concurrent was higher than sequential CRT but the difference was not statistically significant (5.1% vs 2.7%, p = 0.05) (Fig. 2). In the one trial^30^ that compared TRD in sequential CRT with curative RT alone, the rate of TRD was higher in sequential CRT but the absolute difference was small (1.7% vs 0.9%), insufficient for meta-analysis. No trial directly compared palliative with curative RT for TRD.

### Oesophagitis

**Figure 3:**
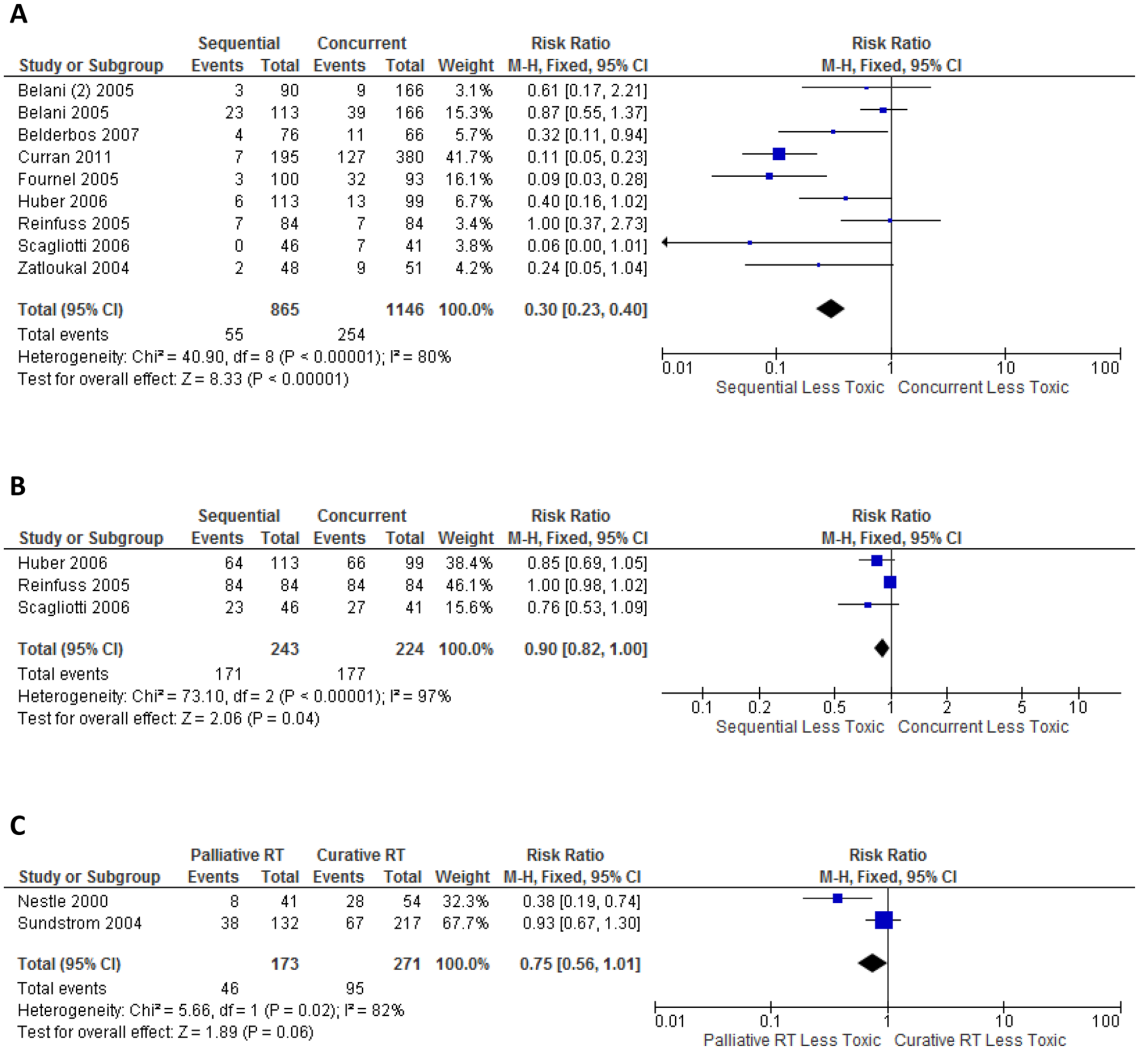
Forest plot showing toxicity risk ratio (RR) for: (**A**) grade ≥ 3 oesophagitis, comparison between sequential versus concurrent chemoradiation; (**B**) any grade oesophagitis, comparison between sequential versus concurrent chemoradiation; (**C**) any grade oesophagitis, comparison between palliative versus curative radiation therapy, generated with Cochrane Review Manager version 5.3

Grade ≥ 3 oesophagitis from concurrent CRT was statistically significantly higher than sequential CRT in 9 trials (22.2% vs 6.4%, p < 0.0001) (Fig. 3A). Any grade oesophagitis from concurrent CRT was also statistically significantly higher than sequential CRT in 3 trials (79.0% vs 70.4%, p = 0.04) (Fig. 3B). 2 trials^48,59^ compared any grade oesophagitis between curative RT and palliative RT, this was higher in curative but the difference was not statistically significant (35.4% vs 26.6%, p = 0.06) (Fig. 3C). Trials were not sufficient for meta-analysis in other comparison groups in assessing grade ≥ 3 or any grade oesophagitis.

### Pneumonitis

**Figure 4:**
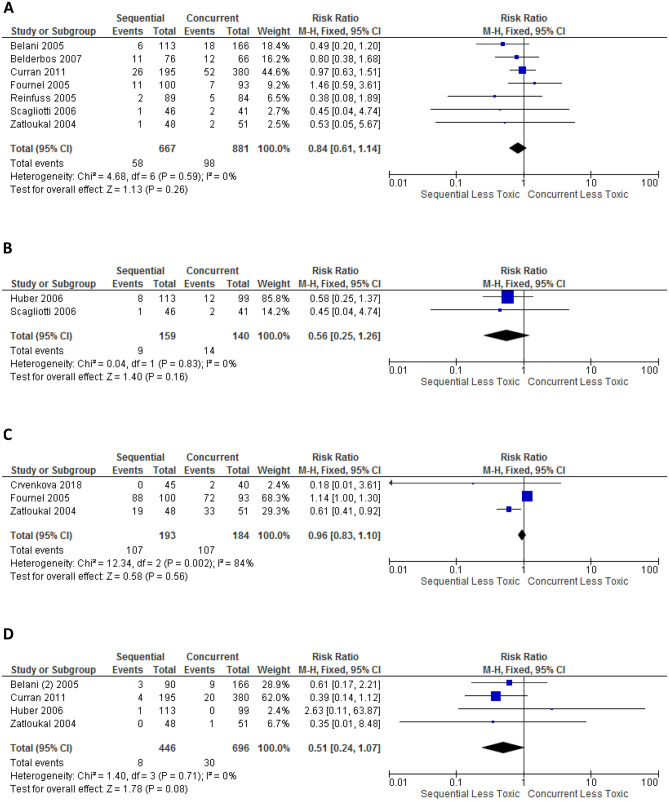
Forest plot showing toxicity risk ratio (RR), comparison between sequential versus concurrent chemoradiation for: (**A**) grade ≥ 3 pneumonitis; (**B**) any grade pneumonitis; (**C**) grade ≥ 3 neutropenia; (**D**) grade ≥ 3 cardiac event, generated with Cochrane Review Manager version 5.3

In the 7 trials that directly compared concurrent with sequential CRT, Grade ≥ 3 pneumonitis from concurrent was higher than sequential CRT, but not statistically significant (11.1% vs 8.7%, p = 0.26) (Fig. 4A). 2 trials^38,53^ directly compared any grade pneumonitis, demonstrating the rate from concurrent CRT was not statistically significantly higher than sequential CRT (10.0% vs 5.7%, p = 0.16) (Fig. 4B). Trials were not sufficient for meta-analysis in other comparison groups in assessing grade ≥ 3 or any grade pneumonitis.

### Neutropenia

Neutropenia was reported in different time intervals following chemotherapy or not specified. Selective reporting of febrile neutropenia was also identified. 3 trials directly compared Grade ≥ 3 neutropenia between concurrent and sequential CRT. Rates from concurrent was higher than sequential CRT, but not statistically significant (58.2% vs 55.4%, p = 0.56) (Fig. 4C). Trials were not sufficient for meta-analysis in assessing any grade neutropenia.

### Cardiac adverse events

4 trials directly compared grade ≥ 3 cardiac events between concurrent and sequential CRT. The rates from concurrent was higher than sequential CRT, but not statistically significant (4.3% vs 1.8%, p = 0.08) (Fig. 4D). Trials were not sufficient for meta-analysis in other comparison groups for grade ≥ 3 or any grade cardiac events.

### Pulmonary fibrosis and myelopathy

Pulmonary fibrosis and radiation myelopathy were poorly reported in the studies. Only 7 trials reported pulmonary fibrosis and 9 trials reported myelopathy across all treatment groups; meta-analysis to compare between groups was not feasible. The rate of pulmonary fibrosis (any grade) was higher in the palliative RT and curative RT arms than the sequential CRT and concurrent CRT. This finding is strongly influenced by a single study by Nestle et al. which reported 100% rate of pulmonary fibrosis based on imaging rather than clinical symptoms.

### Toxicity stratified by stage

**Figure 5:**
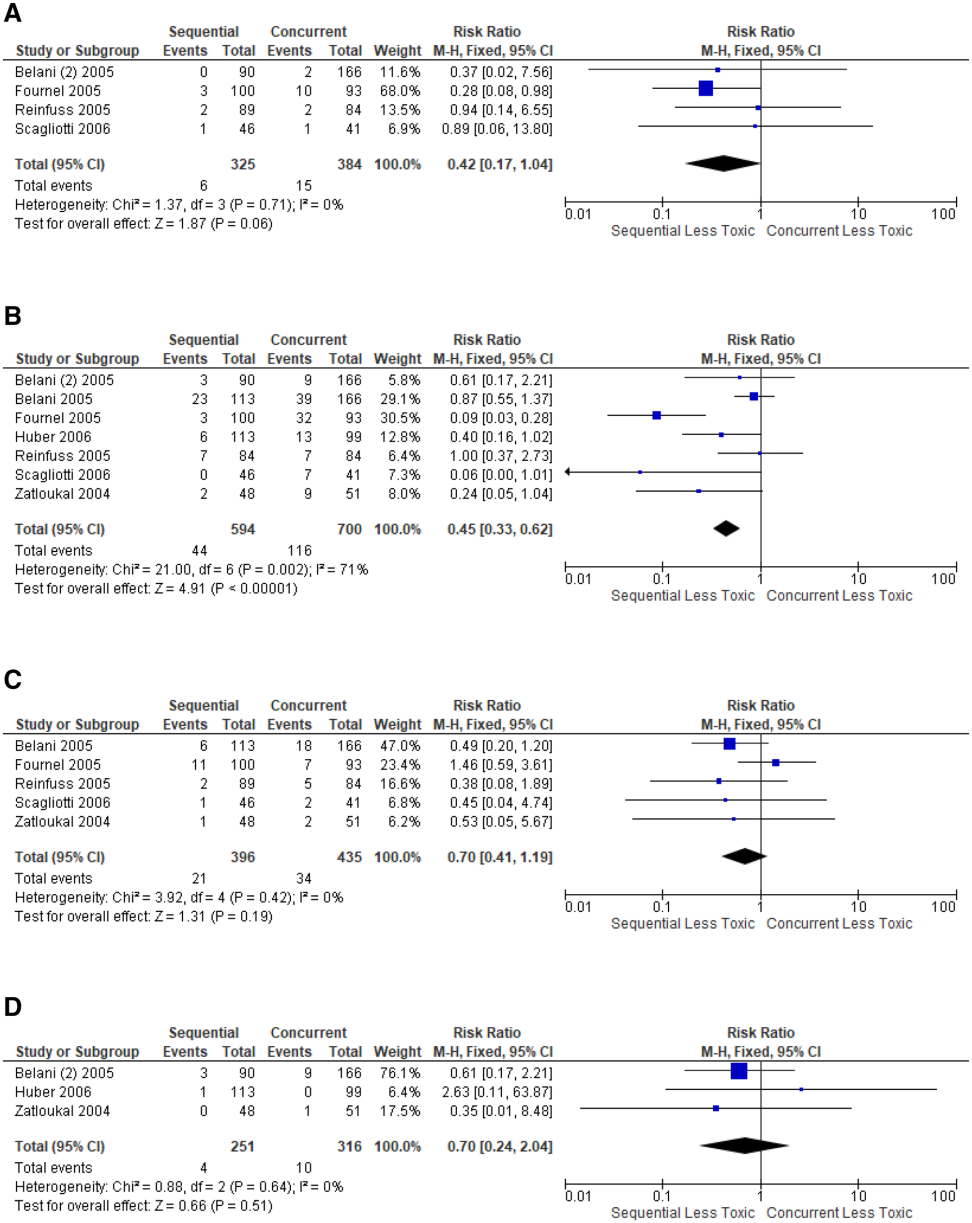
Forest plot showing toxicity risk ratio (RR) for Stage III only studies, comparison between sequential versus concurrent chemoradiation: (**A**) treatment-related deaths; (**B**) grade ≥ 3 oesophagitis; (**C**) grade ≥ 3 pneumonitis; (**D**) grade ≥ 3 cardiac event, generated with Cochrane Review Manager version 5.3

Trials with only stage III NSCLC comparing sequential versus concurrent CRT were analysed (see Fig. 5). The rate of grade ≥ 3 oesophagitis was statistically higher for concurrent CRT (16.6% vs 7.4%, p < 0.0001), whilst the difference in rates of treatment related death, grade ≥ 3 pneumonitis and grade ≥ 3 cardiac events were not statistically significant. Trials were not sufficient for analysis stratified by stage for stages I, II or IV disease.

### Pooled toxicity rates

Overall, the pooled grade ≥ 3 and any grade toxicities rates were lower with sequential compared with concurrent CRT. On pooled comparisons, Grade ≥ 3 (RR 0.75; CI 0.65–0.87) and any grade neutropenia (RR 0.55; CI 0.47–0.64) were significantly less with sequential compared with concurrent CRT. Grade ≥ 3 oesophagitis was also significantly lower with sequential CRT (RR 0.42; CI 0.32–0.54) but not any grade oesophagitis. Any grade cardiac events (RR 0.48; CI 0.23–0.98) and pulmonary fibrosis (RR 0.36; CI 0.20–0.63) were significantly less with sequential compared with concurrent CRT (see Tables 3 and 4).

**Table 3:** Summary table of the pooled grade ≥ 3 toxicity rates.

Pooled toxicity rates (grade ≥ 3)				
	Concurrent chemoradiation	Sequential chemoradiation	Curative RT without chemotherapy	Palliative RT
Treatment related deaths	3.1% (28 trials)	2.4% (9 trials)	1.7% (4 trials)	0% (2 trials)
	RR 0.78 (CI 0.49–1.25)*		N/A	
Oesophagitis (grade ≥ 3)	15.2% (32 trials)	6.4% (9 trials)	0.5% (4 trials)	0.6% (3 trials)
	RR 0.42 (CI 0.32–0.54)*		N/A	
Pneumonitis (grade ≥ 3)	6.6% (28 trials)	6.9% (11 trials)	1.1% (4 trials)	N/A (0 trial)
	RR 1.07 (CI 0.83–1.39)*		N/A	
Neutropenia (grade ≥ 3)	45.7% (19 trials)	34.4% (5 trials)	N/A	N/A
	RR 0.75 (CI 0.65–0.87)*		N/A	
Cardiac (grade ≥ 3)	3.4% (9 trials)	1.9% (5 trials)	2.0% (1 trial)	2.1% (1 trial)
	RR 0.56 (CI 0.29–1.11)*		N/A	
Pulmonary fibrosis (grade ≥ 3)	1.9% (2 trials)	N/A (0 trial)	3.9% (5 trials)	N/A (0 trial)
	N/A		N/A	
Myelopathy (grade ≥ 3)	N/A	N/A	N/A	N/A
	N/A		N/A	

RR* Risk ratio of sequential versus concurrent, *CI* Confidence interval, *N/A* Not applicable.

**Table 4:** Summary table of the pooled any grade toxicity rates.

Pooled toxicity rates (any grade)				
	Concurrent chemoradiation	Sequential chemoradiation	Curative RT without chemotherapy	Palliative RT
Oesophagitis (any grade)	65.6% (12 trials)	69.1% (5 trials)	30.7% (8 trials)	12.2% (6 trials)
	RR 1.05 (CI 0.97–1.13)*		RR 0.40 (CI 0.32–0.50)^#^	
Pneumonitis (any grade)	28.1% (15 trials)	25.3% (5 trials)	39.7% (5 trials)	1.5% (2 trials)
	RR 0.90 (CI 0.74–1.09)*		RR 0.04 (CI 0.02–0.09)^#^	
Neutropenia (any grade)	62.6% (9 trials)	N/A (0 trial)	N/A	N/A
	N/A		N/A	
Cardiac (any grade)	4.4% (4 trials)	2.1% (5 trials)	2.0% (1 trial)	2.1% (1 trial)
	RR 0.48 (CI 0.23–0.98)*		RR 0.68 (CI 0.07–6.37)^#^	
Pulmonary fibrosis (any grade)	25% (2 trials)	9.0% (2 trials)	34.0% (5 trials)	33.8% (2 trials)
	RR 0.36 (CI 0.20–0.63)*		RR 0.99 (CI 0.80–1.24)^#^	
Myelopathy (any grade)	0% (1 trial)	N/A	0.5% (3 trials)	0.2% (6 trials)
	N/A		RR 0.45 (CI 0.06–3.20)^#^	

RR* Risk ratio of sequential versus concurrent, *RR*^*#*^ Risk ratio of palliative versus curative radiation therapy alone, *CI* Confidence interval, *N/A* Not applicable.

Any grade toxicities were also lower with palliative compared with curative radiation alone. On pooled comparisons, any grade oesophagitis (RR 0.40; CI 0.32–0.50) and pneumonitis (RR 0.04; CI 0.02–0.09) were significantly less with palliative compared with curative RT alone (see Tables 3 and 4).

The range of reported grade ≥ 3 oesophagitis was 0 to 41.4% for concurrent CRT and 0 to 20.4% for sequential CRT, whilst any grade oesophagitis ranged between 46.4% and 100% for concurrent CRT and 36.4% to 100% in sequential CRT.

### Risk of bias

Reporting bias were identified with incomplete data and selective reporting of toxicities in most studies, resulting in an overall high risk of bias. Funnel plots were generated to visually assess for publication bias. Symmetrical funnel plots were obtained for comparison groups (> 5 studies) between sequential and concurrent CRT in grade 3 oesophagitis, pneumonitis and treatment-related deaths.

## Discussion

In lung cancer clinical decision making, the consideration of toxicity is essential. As expected, patients receiving palliative RT had lowest toxicity, followed by curative RT alone, sequential and highest with concurrent chemoradiation. The benefit of this review is to provide better estimates of each toxicity effect compared to individual trials.

Acute oesophagitis is one of the main morbidities from lung irradiation. The large differences between individual trials makes it difficult for clinicians to estimate the toxicity in the process of informed consent. The grade ≥ 3 rate with curative RT without chemotherapy is low (0.5%). However, only 4 trials were included, 2 of which included stage I patients only^17,19,25,49^. Although this toxicity is significantly higher with concurrent CRT, it should not be used alone as a factor to preclude concurrent treatment. Oesophagitis can be managed with nutritional support and admission and rarely leads to late stenosis. In addition, IMRT have reduced the incidence of this^65,66^. This difference in oesophagitis rates should be considered as oesophagitis may impact on survival^67^.

Pneumonitis occurs sub-acutely and is the main toxicity of concern as it can result in death. Although the risk of any grade pneumonitis is high for all curative radiotherapy, the risk of Grade 3 + pneumonitis is < 10%. In addition, we found no significant difference between concurrent versus sequential CRT. This suggests that decisions regarding the sequencing of treatment should not be based on the anticipated risk of pneumonitis. However, the increasing use of adjuvant or palliative immunotherapy when combined with prior radiotherapy may potentially increase future pneumonitis risk.

Cardiac toxicity encompasses a range of disorders. Nearly all studies reported cardiac toxicity as a general outcome “cardiac” rather than specifying individual events. The pathophysiology and dose resulting in an event is likely to differ. The risk of grade ≥ 3 toxicity has been correlated with pre-existing cardiac disease and mean heart dose^68^. In breast cancer, Darby et al. found the rates of major coronary events increased linearly with the mean heart dose by 7.4% per Gray^69^. Moreover, data from RTOG 0617 showed heart dose is an independent factor for overall survival^70^. However, a systematic review which includes 3 studies from RTOG 0617 found that heart dose-volume parameters were not consistently associated with survival or cardiac toxicity^71^. Although reduction of heart dose is ideal, any de-escalation of therapy should be carefully weighed against the resulting inferior cure rates^71–74^.

Toxicities for the elderly population are not well established due to the lack of and under-representation in randomised trials. The EORTC and SIOG groups recommended chemotherapy to be considered only in selected fit elderly patients, as the added toxicity may outweigh survival benefit^75^. In this systematic review, there were only two included studies that specified elderly toxicity rates, reflecting the need for randomised trials in this group to aid determine best suitable treatment.

Only one trial included adjuvant immunotherapy and no palliative immunotherapy was used. The studies included treatment with various radiation technique, dose fractionation, including escalated therapy (radiation dose^24,61^ or systemic therapy). Due to changes in radiation technique, older studies (prior to 2000) were not included in this review. Advanced radiation technique such as 3-dimentional compared with 2-dimensional palliative RT to improve conformality can reduce toxicities^76^. Secondary analysis from the RTOG 0617 also confirms that IMRT was associated with lower rates of severe pneumonitis and cardiac doses in locally advanced NSCLC^77^. We did not review toxicities relating to SBRT or the impact of non-chemotherapy systematic therapy.

There are several limitations which are inherent to systematic reviews of randomised trials^78^. Whilst the selected good performance status patients may limit the generalisability, the rates reported in this review may be higher due to escalation of treatment in the experimental arms. On the other hand, real-world patients may also have pre-existing comorbidities and other patient factors which could increase toxicity. This review was unable to analyse toxicity rates based on dose-volume parameters due to insufficient data published in the trials included. Moreover, the pooled rates reported are averages of the toxicity from treatment in different stages. This likely results in an overestimation of risk for those with stage I compared to III disease^79^. The incidence of toxicities reported are crude estimates between the number of patients with toxicities and the total number of patients treated. Actuarial estimates provides a more accurate determination toxicities prevalence^80^. The findings from this review should be interpreted with some caution.

We included randomised trials as protocols with prospective data generally provides better-quality toxicity data. In concordance with Sivendran et al. on adverse event reporting in cancer clinical trial publications, we identified selectivity and heterogeneity with reporting toxicities in trials^81^. The quality of studies examined ranged from low to high, contributed by reporting bias. There was variation in timing of reported toxicities, different toxicity grading criteria used and limited studies on quality of life. This highlights the need for trials to report reliable toxicity data, ideally under standardised criteria and in conjunction with the Consolidated Standards of Reporting Trials (CONSORT) recommendations^82^.

To the best of our knowledge this is the only available review of toxicity data in recent trials that compares and provides estimates of palliative radiotherapy, curative radiotherapy, sequential and concurrent chemoradiotherapy regimens. The only statistically significant difference between treatment regimens was the rate of oesophagitis with concurrent versus sequential CRT. This information is clinically useful and should be considered by clinicians and patients when weighing up the established survival benefits with the toxicity of the different treatment options.

## Supplementary Information

Supplementary Information.
