# Supplementary material for: A systematic review and meta-analysis of treatment-related toxicities of curative and palliative radiation therapy in non-small cell lung cancer
Source: Sci Rep. 2021 Mar 15;11:5939. doi: 10.1038/s41598-021-85131-7 (PMC7971013; doi:10.1038/s41598-021-85131-7)
Supplement: Supplementary file 1 — Supplementary Information. [file 41598_2021_85131_MOESM1_ESM.docx]

# A Systematic Review and Meta-analysis of Treatment-Related Toxicities of Curative and Palliative Radiation Therapy in Non-Small Cell Lung Cancer

**Authors:** M Or^1^; B Liu^1^; J Lam^2^, S Vinod^3,4^; W Xuan^4,5^; R Yeghiaian-Alvandi^1^; E Hau^1^

**Affiliations:** ^1^Crown Princess Mary Cancer Centre, Westmead Hospital, Sydney, NSW, Australia, ^2^Northern Sydney Cancer Centre, Royal North Shore Hospital, Sydney, NSW, Australia, ^3^Cancer Therapy Centre, Liverpool Hospital, Liverpool, NSW, Australia, ^4^South Western Sydney Clinical School, University of New South Wales, NSW, Australia, ^5^Ingham Institute for Applied Medical Research, Liverpool, NSW, Australia.

**Corresponding Author**

Dr Michelle Or

Department of Radiation Oncology
The Crown Princess Mary Cancer Centre, Westmead Hospital, Westmead Sydney NSW 2145

Telephone +61 2 8890 5200
Fax +61 2 8890 8567

michelle.pt.or@gmail.com

# **Appendices**

## **Appendix A.1 Data Collection Template**

**Data collection template (Page 1/2)**

**Reviewer and date: Circle: Include / Exclude**

| Report Title |  | |  |
| --- | --- | --- | --- |
| Trial name/code |  | | |
| First author |  | **Year of publication** | |
| Source/Journal |  | | |
| Publication Type | **Journal** | **Abstract** | **Other (specify)** |
| Language | **English** | | **Other (specify)** |
| Type of study | **Randomised** | | **Other (specify)** |
| Radiotherapy | **Curative** | | |
|  | **Concurrent Chemo-radiation** | **Sequential Chemo-radiation** | **Radiation therapy alone** |
|  | 60-70Gy in 30-35# | 50-55Gy in 20# | HFRT schemes, CHART |
|  | **Palliative radiation therapy alone** | | |
|  | 30-36Gy in 10-12# | 20Gy in 5#  16-17Gy in 2# | 8-10Gy in 1# |

Abbreviations: Gy – Gray; # - fraction; HFRT – Hypofractionated radiation therapy; CHART – Continuous, hyperfractionated, accelerated radiotherapy.

## **Appendix A.2 Data Collection Template**

**Data collection template (Page 2/2)**

| Types of outcomes |  | Time of reporting | | | | | | |
| --- | --- | --- | --- | --- | --- | --- | --- | --- |
|  |  | **1m** | **3m** | **6m** | **1y** | **2y** | **3y** | **Other -specify** |
|  | **Treatment-related deaths** |  |  |  |  |  |  |  |
|  | **Treatment discontinuation** |  |  |  |  |  |  |  |
|  | **Toxicity reporting system:**  **CTCAE / RTOG / Others** | | | **Specify version:**  **Version -** | | | | |
|  | **Acute pneumonitis G1** |  |  |  |  |  |  |  |
|  | **Acute pneumonitis G2** |  |  |  |  |  |  |  |
|  | **Acute pneumonitis G3** |  |  |  |  |  |  |  |
|  | **Acute pneumonitis G4** |  |  |  |  |  |  |  |
|  | **Acute oesophagitis G1** |  |  |  |  |  |  |  |
|  | **Acute oesophagitis G2** |  |  |  |  |  |  |  |
|  | **Acute oesophagitis G3** |  |  |  |  |  |  |  |
|  | **Acute oesophagitis G4** |  |  |  |  |  |  |  |
|  | **Cardiac G1** |  |  |  |  |  |  |  |
|  | **Cardiac G2** |  |  |  |  |  |  |  |
|  | **Cardiac G3** |  |  |  |  |  |  |  |
|  | **Cardiac G4** |  |  |  |  |  |  |  |
|  | **Pulmonary fibrosis G1** |  |  |  |  |  |  |  |
|  | **Pulmonary fibrosis G2** |  |  |  |  |  |  |  |
|  | **Pulmonary fibrosis G3** |  |  |  |  |  |  |  |
|  | **Pulmonary fibrosis G4** |  |  |  |  |  |  |  |
|  | **Myelopathy G1** |  |  |  |  |  |  |  |
|  | **Myelopathy G2** |  |  |  |  |  |  |  |
|  | **Myelopathy G3** |  |  |  |  |  |  |  |
|  | **Myelopathy G4** |  |  |  |  |  |  |  |
|  | **Febrile Neutropenia G1** |  |  |  |  |  |  |  |
|  | **Febrile Neutropenia G2** |  |  |  |  |  |  |  |
|  | **Febrile Neutropenia G3** |  |  |  |  |  |  |  |
|  | **Febrile Neutropenia G4** |  |  |  |  |  |  |  |

Abbreviations: CTCAE – Common Terminology Criteria for Adverse Events; RTOG – Radiation Therapy Oncology Group; G – Grade; m – month; y – year.
